# Comparison of bacterial microbiota of the predatory mite *Neoseiulus cucumeris* (Acari: Phytoseiidae) and its factitious prey *Tyrophagus putrescentiae* (Acari: Acaridae)

**DOI:** 10.1038/s41598-017-00046-6

**Published:** 2017-01-31

**Authors:** Apostolos Pekas, Eric Palevsky, Jason C. Sumner, M. Alejandra Perotti, Marta Nesvorna, Jan Hubert

**Affiliations:** 1 Research & Development Department, Biobest Belgium N. V., Ilse Velden 18, Westerlo B-2260 Belgium; 2 Department of Entomology, Newe-Ya’ar Research Center, Agricultural Research Organization, Ministry of Agriculture, P.O. Box 1021 Ramat Yishay, IL-30095 Israel; 3 SASA (Science and Advice for Scottish Agriculture), 1 Roddinglaw Road, Edinburgh, EH12 9FJ UK; 4 Evolutionary Biology Section, School of Biological Sciences, University of Reading, Reading, RG6 6AS UK; 5 Crop Research Institute, Drnovska 507/73, Prague 6-Ruzyne, CZ-161 06 Czechia Czech Republic

## Abstract

*Neoseiulus cucumeris* is a predatory mite used for biological control of arthropod pests. Mass-reared predators are fed with factitious prey mites such as *Tyrophagus putrescentiae*. Although some information on certain endosymbionts of *N. cucumeris* and *T. putrescentiae* exists, it is unclear whether both species share bacterial communities. The bacterial communities in populations of predator and prey mites, as well as the occurence of potential acaropathogenic bacteria were analyzed. The comparisons were based on the following groups: (i) *N. cucumeris* mass-production; (ii) *N. cucumeris* laboratory population with disease symptoms; (iii) *T. putrescentiae* pure populations and; (iv) *T. putrescentiae* from rearing units of *N. cucumeris*. Only 15% of OTUs were present in all samples from predatory and prey mite populations (core OTUs): the intracellular symbionts *Wolbachia*, *Cardinium*, plus other *Blattabacterium*-like, *Solitalea*-like, and *Bartonella*-like symbionts. Environmental bacteria were more abundant in predatory mites, while symbiotic bacteria prevailed in prey mites. Relative numbers of certain bacterial taxa were significantly different between the microbiota of prey mites reared with and without *N. cucumeris*. No significant differences were found in the bacterial communities of healthy *N. cucumeris* compared to *N. cucumeris* showing disease symptoms. We did not identify any confirmed acaropathogenic bacteria among microbiota.

## Introduction

Phytoseiid mites (Acari: Phytoseiidae) are amongst the most important predators used in plant protection against arthropod pests such as spider mites, whiteflies and thrips^1–3^. Many species of commercially available phytoseiid mites are mass reared using astigmatid mites (Acari: Astigmata) as factitious prey^4,5^. Like many other arthropod species, predatory and prey mites are closely associated with symbiotic and pathogenic bacteria that may have variable yet critical impact on several fitness parameters of their arthropod hosts^6,7^. Diseases and/or reproductive disorders associated with endosymbiotic bacteria can have devastating effects on the mass-rearing of predatory mites^8^. Similarly, poor quality of prey mites due to infestation with pathogenic bacteria will compromise the production of predatory mites^9,10^. Until recently, studies on microbiota relied on the use of molecular markers targeting specific endosymbiont species. Recent advances in molecular biology and bioinformatics allow for the rapid screening of the whole microbiome and provide useful insights into the bacterial communities of predatory and prey mites. Given the prominent role of phytoseiid mites as biological control agents, screening the symbiotic and pathogenic bacterial community and establishing an association with phenotypic traits can potentially impact rearing protocols used by the biocontrol industry^11^. Moreover, the high densities of mites in mass rearing conditions and reports of horizontal bacterial transmission between trophic levels^12^ suggest these environments offer an ideal setting for the comparative study of the bacterial microbiota of predatory and prey mites.

Previous studies reported the presence of endosymbiotic and pathogenic bacteria in phytoseiid mites^8,9^. *Metaseiulus occidentalis* (Nesbitt) is probably the best studied species^7^. Several bacteria were detected including: the pathogenic *Serratia marcescens*; two *Rickettsia*-like bacteria; the gut symbionts *Enterobacter* and *Bacteroidetes*; and the endosymbionts *Wolbachia* and *Cardinium*^7^. Enigl and Schausberger^13^ screened several phytoseiid species for the presence of *Wolbachia*, *Cardinium* and *Spiroplasma*, they found *Cardinium* in *Euseius finlandicus* (Oudemans) and *Spiroplasma* in *Neoseiulus californicus* (McGregor). Similarly, *Wolbachia* and *Cardinium* showed different patterns of infection in the phytoseiid mite *N. paspalivorus* (De Leon) depending on geographic origin^14^. Interestingly, the observed postzygotic reproductive incompatibility among populations was associated with the presence of endosymbiotic bacteria. Gols *et al.*^15^ discovered the bacterium *Acaricomes phytoseiuli* in several commercial populations of the phytoseiid mite *Phytoseiulus persimilis* (Athias-Henriot). The infected predatory mites exhibited lower fecundity and longevity and reduced attraction to plant volatiles induced by spider mites. This was designated as non-responding syndrome and rendered infected *P. persimilis* populations unsuitable for effective control of spider mites.

Regarding prey astigmatid mites, sequencing of the 16S rRNA gene revealed that the bacterial communities in *Acarus siro* L., *Lepidoglyphus destructor* (Schrank) and *Tyrophagus putrescentiae* (Schrank) were formed by ingested bacteria. These included: *Bacillus*, *Staphylococcus* and *Kocuria* spp.; the gut bacteria Enterobacteriaceae and *Bartonella*-like bacteria; endosymbiotic bacteria such as *Cardinium*; and/or entomopathogenic bacteria *Xenorhabdus* and *Photorhabdus*^16,17^.

*Neoseiulus cucumeris* (Oudemans) is one of the most widely employed predators in augmentative biological control programs against thrips species such as *Frankliniella occidentalis* (Pergande) and *Thrips tabaci* Lindeman (Thysanoptera: Thripidae) mostly in protected crops^18–20^. For the mass-rearing of *N. cucumeris*, the acaricid mite *T. putrescentiae* is used as factitious prey^21^. While some information on certain endosymbionts of *N. cucumeris* and *T. putrescentiae*^2,3^ exists there is no information regarding the intrinsic microbiota of these species, or the effects of predatory-prey mite interactions on the ecology of these microbiota. Although mites are kept at high densities in mass rearing units it is still unclear whether both species share bacterial communities due to horizontal transfer via predation, contact or the feces^6^. The current study, to our knowledge, is the first direct comparison of bacterial microbiota from Phytoseiidae mites and their factitious prey mites.

Understanding the composition of the bacterial microbiota in both *N. cucumeris* and *T. putrescentiae* will allow for detecting pathogenic bacteria in the mass-rearing systems and perhaps offer opportunities for the manipulation of the bacterial community to improve predatory mite health. Both of the above mentioned scenarios can potentially have a substantial impact on biological control. In the present study we use Illumina sequencing of the 16S rRNA gene region and bioinformatics tools to (i) characterize the entire bacterial microbiota (ii) identify potentially pathogenic bacteria and iii) compare bacterial microbiota to examine the effects of predator-prey interactions and different rearing conditions on microbial ecology.

## Results

### Bacterial microbiota characterization

**Figure 1:**
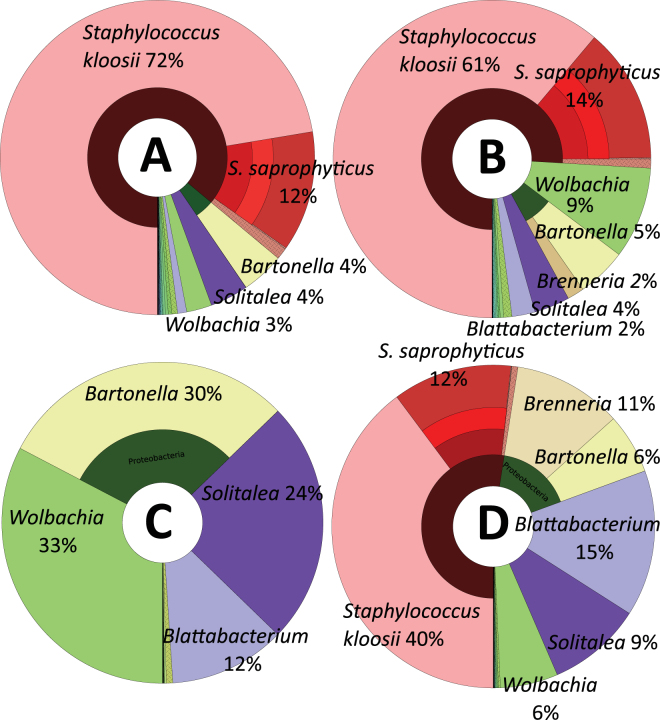
The Krona projections of bacterial taxa found in the samples of predator (*N. cucumeris*) and prey (*T. putrescentiae*) mites. (**A**) Predatory mite (*N. cucumeris*) from mass-production population; (**B**) predatory mite from a laboratory population with disease symptoms; (**C**) the prey mite (*T. putrescentiae*) population from laboratory culture without the presence of predatory mite (Tyro pure), (**D**) prey mite from culture with the presence of predatory mite in mass rearing.

**Figure 2:**
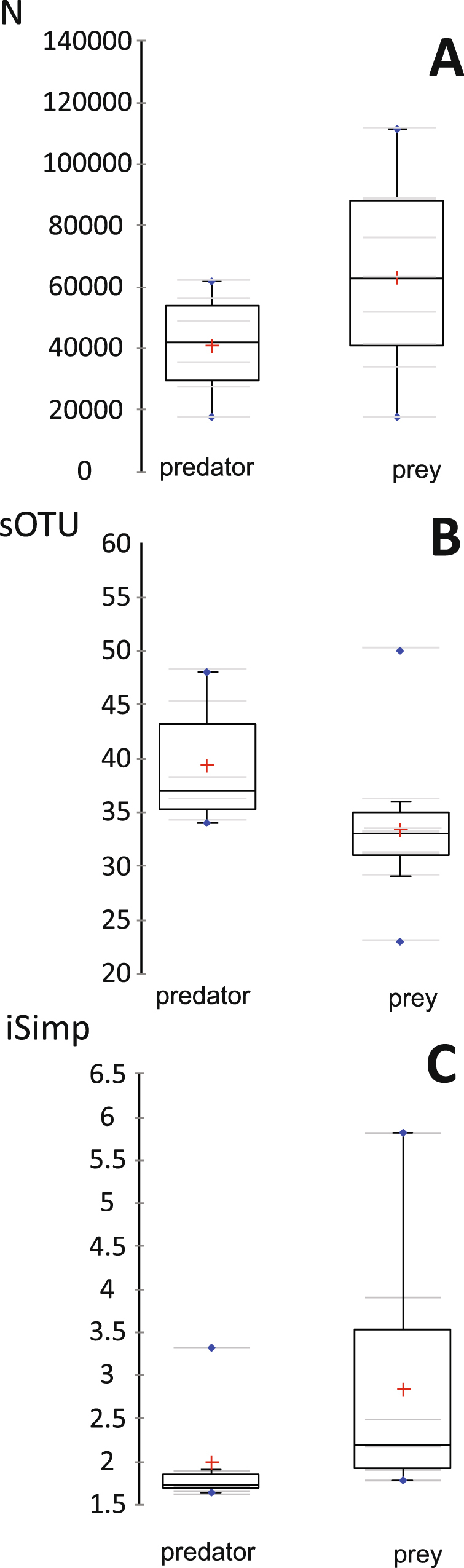
Comparisons of beta-diversity between bacterial microbiota from *N. cucumeris* compared to *T. putrescentiae*. (**A**) Number of sequences analyzed across samples; (**B**) Species Richness, number of species-level OTUs across samples; (**C)** Inverse Simpson Diversity Index comparing predatory vs. prey mites (see Table 1 for description of samples).

The 16S rRNA gene libraries included 818,413 sequences classified in 75 OTUs. The proportion of bacterial taxa in the groups of predatory and prey mites were visualized in Krona projection (Fig. 1). The minimal number of reads was 17,613 (Fig. 2A). No known acaropathogenic bacteria were identified in the prey or predatory mites either from the mass rearing or the laboratory population with disease symptoms (Table S1).

### Bacterial microbiota comparisons: *N. cucumeris* (predator) vs. *T. putrescentiae* (factitious prey)

**Figure 3:**
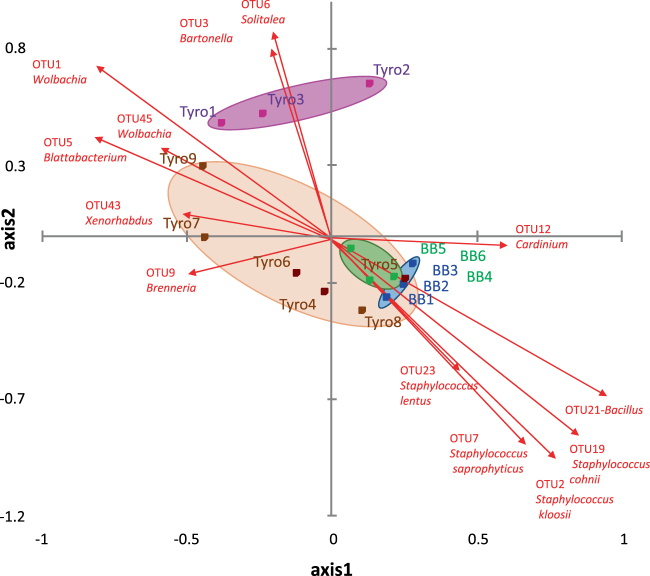
Principal coordinate analyses (PCoA) of microbiota in the samples of predatory (*N. cucumeris*) and prey (*T. putrescentiae*) mites. The microbiome was analyzed using Bray-Curtis dissimilarity matrix; OTUs responsible for significant differences between microbiotas are represented by arrows (calculated via Pearson correlation coefficient). BB1-3 = *N. cucumeris* (laboratory); BB4-6 = *N. cucumeris* (mass-reared); Tyro1-3 = *T. putrescentiae* laboratory culture without the presence of predatory mite (pure) and Tyro4-9 = from *N. cucumeris* mass-rearing The samples are described in Table 1, the OTUs are identified in Table S1.

Total numbers of OTUs found in predatory mites compared to prey mites were marginally non-significant (Fig. 2B). The Inverse Simpson index was 1.5 times higher in prey compared to predatory mite samples (U_(1,14)_ = 8, P = 0.029) (Fig. 2C). Bray-Curtis dissimilarity index showed higher dissimilarity in bacterial composition within prey mite samples than within the predatory mite samples. There were significant differences between the bacterial composition of prey mites compared to predator mites (i.e. factor 1, Table S2) (two-way PERMANOVA, F = 0.947, P = 0.011). There was also significant difference between the populations of mites (i.e. factor 2, Table S2) (two-way PERMANOVA, F = 0.66, P = 0.012), but the interaction between mite species and different population factors was not significant (F = −2.19, P = 0.999). When analyzed using Jaccard similarity matrix, the results were in agreement with previous analyses. Species of mite was a significant factor affecting the bacterial diversity of microbiota (F = 0.245, P = 0.045), as was the population that they originated from (F = 0.303, P < 0.001) but their interaction was not significant (F = −1.89, P = 0.972). Sample dissimilarity was visualized by PCoA; the first axis explained 56% and the second axis 15% of variability in the data set (Fig. 3). Microbiota of predator and prey mites formed two distinct clustered when represented by PCoA. Distances between OTUs along the first axis can be attributed to differences between predator and prey microbiota, with the exception of Tyro5 and Tyro8 (*T. putrescentiae* from *N. cucumeris* mass rearing units) where the bacterial composition was similar to that of the predatory mites. The following bacterial taxa were associated with prey mites: *Solitalea*-like (OTU6), *Bartonella*-like (OTU3), *Wolbachia* (OTU1 and 45), *Blattabacterium*-like (OTU5), *Brenneria* (OTU9) and *Xenorhabdus* (OTU43). The following taxa were associated with predatory mites: *Cardinium* (OTU12), *Bacillus* (OTU21) and *Staphylococcus* (OTUs2, 7, 19 and 23).

**Figure 4:**
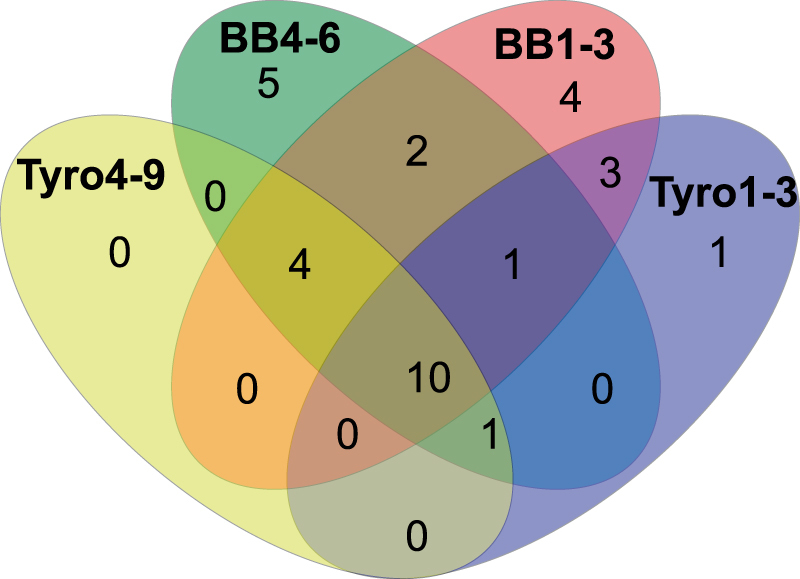
Qualitative comparison of shared and unique bacterial taxa belonging to predatory and prey mite microbiota by Venn diagram. The compared samples include predatory mites (*Neoseiulus cucumeris*) from the mass-production population (BB1-3) and the population with disease symptoms (BB4-6); the prey mites (*Tyrophagus putrescentiae*) from pure laboratory cultures without predators (Tyro1-3) and from the mass rearing production population with the presence of predators (Tyro4-9). The diagram was constructed from the core species per samples. The shared OTUs among all samples were the following taxa ordered by decreasing relative abundance: *Staphylococcus kloosii* (OTU2), *Wolbachia* (OTU1), *Blattabacterium-*like (OTU5), *Bartonella*-like (OTU3), *Solitalea*-like (OTU6), *Staphylococcus saprophyticus* (OTU7), *Staphylococcus cohnii* (OTU19), *Bacillus cereus* (OTU21), *Cardinium* (OTU12), *Brevibacterium siliguriense* (OTU16). *Brenneria* (OTU9), *Staphylococcus lentus* (OTU23), *Kocuria koreensis* (OTU25) and *Xenorhabdus innexi* (OTU43) were shared by predatory mites (BB1-3 and BB4-6) and prey mites from the mass rearing production population (Tyro4-9). *Wolbachia* (OTU45) was shared by predatory mites (BB1-3 and BB4-6) and prey mites from pure laboratory cultures without predators (Tyro1-3). *Arthrobacter* (OTU47) was shared by predatory mite population with disease symptoms (BB4-6) and both populations of the prey mites (Tyro1-3 and Tyro4-9).

Venn diagrams show that 11 OTUs were shared by prey mites and 16 OTUs by predatory mites. Altogether 10 core OTUs (15% of the total OTUs) were present in all samples of both predatory and prey mites (Fig. 4). This group contained: endosymbiotic bacteria such as *Wolbachia* (OTU1), *Cardinium* (OTU12), *Bartonella*-like (OTU3), *Blattabacterium*-like (OTU5), *Solitalea*-like (OTU6) and environmental bacteria *Brevibacterium* (OTU16), *Staphylococcus* spp. (OTUs2, 7 and 9) and *Bacillus cereus* (OTU21).

From the 75 OTUs analyzed by METASTATS, 10 OTUs had higher relative abundance in predatory mites compared with prey mites (Table 1). OTUs associated with predatory mites were *Brevibacterium* (OTU18), *Staphylococcus* (OTU2 and OTU19), *Bacillus* (OTU21), *Kocuria* (OTU25 and OTU39), *Stenotrophomonas* (OTU52), *Chryseobacterium* (OTU84) and *Pantoea* (OTU83). Only 2 OTUs were most abundant in prey mites the symbiotic/parasitic bacteria *Blattabacterium*-like (OTU5) and *Solitalea-*like (OTU6) (Table 1). The remaining 63 OTUs were not influenced by the prey/predator mites (i.e. factor 1, Table S2).

**Table 1:** The list of OTUs in the samples of predatory (*Neoseiulus cucumeris*) and prey (*Tyrophagus putrescentiae*) mites and the results of Random Forest and METASTATS analyses describing the means of relative abundance of OTUs (%) for the samples of predatory and prey mites.

OTU_97_		GenBank identification	Random forest		METASTATS		
aOTU	OTU ID	Taxon	predator/prey	populations	prey	predator	p-value
					mean ± standard error		
OTUs presented in all samples							
456731	OTU2	*Staphylococcus kloosii* (99)	0.1	0.1	26.62 ± 10.04	66.75 ± 4.01	**0.001**
180511	OTU1	*Wolbachia* (97)		0.02	14.74 ± 6.77	5.97 ± 2.47	0.267
122903	OTU5	*Blattabacterium*-like	0.1		13.66 ± 3.92	1.64 ± 0.48	**0.003**
101995	OTU3	*Bartonella queenslandensis* (95)	0.08	0.06	14.06 ± 5.90	4.72 ± 0.29	0.102
71643	OTU6	*Solitalea*-like	0.17	0.03	14.47 ± 3.62	3.77 ± 0.59	**0.004**
64153	OTU7	*Staphylococcus saprophyticus* (99)	0.13	0.13	7.97 ± 2.56	13.06 ± 1.80	0.091
5593	OTU19	*Staphylococcus cohnii* (99)	0.16	0.01	0.32 ± 0.08	0.69 ± 0.06	**0.000**
2402	OTU21	*Bacillus cereus* (99)	0.07	0.04	0.21 ± 0.04	0.37 ± 0.04	**0.004**
1730	OTU12	*Cardinium*	0.06	0.06	0.12 ± 0.03	0.1 ± 0.03	0.142
1569	OTU16	*Brevibacterium siliguriense* (97)	0.09	0.07	0.09 ± 0.04	0.34 ± 0.16	0.148
OTUs presented in the samples of predators and prey from mass rearing							
40567	OTU9	*Brenneria salicis* (91)	0.13	0.15	7.28 ± 3.74	0.87 ± 0.55	0.075
563	OTU23	*Staphylococcus lentus* (99)	0.09	0.07	0.04 ± 0.01	0.10 ± 0.02	**0.007**
366	OTU25	*Kocuria koreensis* (96)	0.32	0.14	0.01 ± 0.003	0.09 ± 0.04	**0.015**
350	OTU43	*Xenorhabdus innexi* (92)	0.07	0.02	0.03 ± 0.01	0.02 ± 0.004	0.491
OTUs presented in the samples of predators and prey from laboratory							
133	OTU45	*Wolbachia* (94)	0.06	0.02	0.01 ± 0.002	0.02 ± 0.01	0.283
OTUs presented in the samples of sick predator and both groups of prey							
78	OTU47	*Arthrobacter russicus* (91)	0.06	0.01	0.01 ± 0.003	0.02 ± 0.01	0.492
OTUs presented in the two groups of samples							
389	OTU26	*Brevibacterium iodinum* (98)	0.06	0.02	0.03 ± 0.01	0.09 ± 0.04	0.188
241	OTU39	*Kocuria koreensis* (99)	0.07	0.04	0.02 ± 0.01	0.06 ± 0.02	**0.028**
152	OTU61	*Pseudomonas monteilii* (99)	0.13	0.04	0.01 ± 0.003	0.08 ± 0.05	0.091
142	OTU35	*Moraxella osloensis* (99)	0.1	0.04	0.01 ± 0.003	0.05 ± 0.03	0.073
100	OTU32	*Bartonella coopersplainsensis* (95)	0.07	0.02	0.01 ± 0.003	0.01 ± 0.001	0.943
66	OTU38	*Bartonella rattaustraliani* (98)	0.07	0.03	0.004 ± 0.001	0.01 ± 0.002	0.751
OTUs presented in one group of samples							
953	OTU11	*Wolbachia* (97)	0.1	0.05	0.02 ± 0.01	0.17 ± 0.11	0.189
565	OTU18	*Brevibacterium ocean*i (99)	0.1		0.01 ± 0.01	0.16 ± 0.07	**0.023**
317	OTU22	*Corynebacterium variabile* (97)	0.08	0.1	0.03 ± 0.01	0.06 ± 0.03	0.271
166	OTU31	*Propionibacterium acnes* (99)	0.08	0.02	0.03 ± 0.02	0.02 ± 0.01	0.675
105	OTU55	*Acinetobacter lwoffii* (97)	0.16		0.001 ± 0.001	0.05 ± 0.03	0.100
99	OTU53	*Lactococcus lactis* (99)	0.08	0.01	0.01 ± 0.003	0.02 ± 0.02	0.718
83	OTU48	*Leuconostoc gasicomitatum* (99)	0.07	0.03	0.01 ± 0.01	0.01 ± 0.01	0.779
81	OTU52	*Stenotrophomonas maltophilia* (99)	0.07		0.01 ± 0.002	0.02 ± 0.01	**0.027**
61	OTU96	*Tsukamurella paurometabola* (99)	0.13	0.1	0.01 ± 0.01	0.01 ± 0.01	0.869
37	OTU91	*Streptococcus thermophilus*(99)	0.11	0.08	—	0.02 ± 0.01	0.122
682	OTU13	*Wolbachia* (99)	0.08		0.001 ± 0.001	0.14 ± 0.14	0.457
149	OTU50	*Pseudomonas poae* (99)	0.03		0.01 ± 0.01	0.003 ± 0.002	0.622
128	OTU51	*Alcaligenes faecalis* (99)	0.08	0.01	0.01 ± 0.01	0.02 ± 0.02	0.868
115	OTU34	*Microbacterium indicum* (97)	0.08	0.02	0.03 ± 0.03	0.03 ± 0.02	0.980
92	OTU60	*Wautersiella falsenii* (97)	0.11		0.004 ± 0.003	0.02 ± 0.02	0.237
73	OTU36	*Paracoccus chinensis* (99)	0.04		—	0.03 ± 0.03	0.436
73	OTU42	*Prevotella paludivivens* (97)	0.07	0.02	0.001 ± 0.001	0.03 ± 0.03	0.461
72	OTU62	*Cloacibacterium rupense* (96)	0.04	0.01	0.01 ± 0.01	—	0.410
70	OTU49	*Acinetobacter baumannii* (99)	0.17	0.04	—	0.04 ± 0.02	0.077
69	OTU40	*Corynebacterium nuruki* (99)	0.07	0.02	0.01 ± 0.01	0.02 ± 0.01	0.656
67	OTU70	*Pseudochrobactrum asaccharolyticum* (99)	0.13	0.01	0.01 ± 0.01	0.001 ± 0.001	0.737
61	OTU87	*Sphingobacterium multivorum* (99)	0.13	0.04	0.01 ± 0.01	0.001 ± 0.001	0.637
58	OTU69	*Corynebacterium vitaeruminis* (99)	0.11	0.02	0.01 ± 0.01	—	0.242
57	OTU63	*Acinetobacter johnsonii* (99)	0.13	0.05	—	0.03 ± 0.02	0.256
57	OTU94	*Staphylococcus aureus* (99)	0.13	0.07	0.01 ± 0.01	0.01 ± 0.01	0.711
50	OTU57	*Paenochrobactrum glaciei* (99)	0.09	0.01	0.01 ± 0.01	—	0.129
47	OTU77	*Ralstonia insidiosa* (99)	0.11	0.06	0.01 ± 0.01	0.01 ± 0.01	0.883
45	OTU71	*Leclercia adecarboxylata* (99)	0.1	0.02	0.001 ± 0.001	0.03	0.295
33	OTU103	*Escherichia coli* (99)	0.08	0.01	0.001 ± 0.001	0.001 ± 0.001	1.000
32	OTU56	*Lactococcus chungangensis* (97)	0.08		—	0.01 ± 0.01	0.229
31	OTU79	*Delftia tsuruhatensis* (99)	0.13	0.05	0.001 ± 0.001	0.01 ± 0.01	0.323
30	OTU82	*Acinetobacter radioresistens* (99)	0.1	0.02	0.001 ± 0.001	0.01 ± 0.01	0.370
27	OTU95	*Leucobacter denitrificans* (97)	0.13	0.05	0.01 ± 0.01	0.001 ± 0.001	0.269
25	OTU54	*Pseudomonas caeni* (99)	0.07	0.01	0.003 ± 0.003	0.01 ± 0.01	0.562
25	OTU64	*Brevundimonas bullata* (99)	0.12	0.03	0.003 ± 0.003	0.01 ± 0.005	0.591
24	OTU101	*Brevibacterium siliguriense* (96)	0.14		—	0.01 ± 0.01	0.084
22	OTU68	*Chryseobacterium bernardetii* (94)	0.04	0.01	0.001 ± 0.001	—	0.520
22	OTU74	*Sphingobacterium faecium* (99)	0.06	0.02	—	0.01 ± 0.01	0.386
20	OTU102	*Alcanivorax dieselolei* (94)		0.01	0.003 ± 0.003	0.001 ± 0.001	0.412
18	OTU104	*Corynebacterium singulare* (99)	0.04		—	0.01 ± 0.01	0.102
18	OTU72	*Finegoldia magna* (99)	0.09	0.05	—	0.01 ± 0.01	0.211
14	OTU88	*Rickettsia bellii* (99)	0.08		0.001 ± 0.001	—	0.155
13	OTU75	*Acidovorax radicis* (99)	0.05	0.03	—	0.01 ± 0.01	0.115
13	OTU92	*Paenibacillus hordei* (97)		0.04	0.01 ± 0.01	—	0.410
12	OTU84	*Stenotrophomonas rhizophila* (99)	0.1	0.03	—	0.01 ± 0.01	0.292
12	OTU89	*Buchnera aphidicola* (97)	0.07	0.04	0.001 ± 0.001	0.01 ± 0.01	0.180
10	OTU80	*Paracoccus marinus* (99)	0.09	0.05	—	0.001 ± 0.001	0.160
10	OTU83	*Pantoea calida* (94)	0.07	0.04	0.001 ± 0.001	0.005 ± 0.005	**0.041**
10	OTU93	*Afipia birgiae* (99)	0.07	0.07	0.001 ± 0.001	0.001 ± 0.001	0.395
9	OTU81	*Chryseobacterium balustinum (97)*	0.06	0.02	—	0.001 ± 0.001	**0.026**
7	OTU86	*Lactobacillus paracollinoide*s (99)	0.11	0.05	0.001 ± 0.001	0.001 ± 0.001	0.309
6	OTU97	*Anaerococcus senegalensis* (99)	0.04	0.01	—	0.001 ± 0.001	0.064
4	OTU65	*Phyllobacterium myrsinacearum* (99)	0.02	0.01	0.001 ± 0.001	0.001 ± 0.001	1.000

Supplementary Table S1 provides the extensive identification of OTUs. The random forest analyze was calculated for predatory/prey and populations as the factors (see Table S2), separately. The forest error rates were 0.33 and 0.53 respectively, aOTU - total number of sequnces in dataset, P-values < 0.05 are indicated by bold.

### Differences in bacterial microbiota within populations of predatory and prey mites

There were no significant differences in the bacterial composition between lab-reared (showing disease symptoms) or mass-reared populations of *N. cucumeris* (one way PERMANOVA, F = 2.312, P = 0.198). This was supported by their proximity on the axes of the principle coordinate analysis (PCoA) (Fig. 3). The random forest algorithms (forest error rate = 0.66) indicated the following OTUs as the most important for differences: *Stenotrophomonas rhizophila* (OTU84) *Lactococcus chungangensis* (OTU56), *Lactococcus lactis* (OTU53), *Leclercia adecarboxylata* (OTU71). These were OTUs present only in healthy predator populations. The Venn diagram showed that 8 OTUs were found only in the populations of mites with disease symptoms: *Wolbachia* (OTU11), *Corynebacterium variabile* (OTU22), *Brevibacterium oceani* (OTU11), *Stenotrophomonas maltophilia* (OTU52) (Fig. 4, Table 1).

Significant differences were detected between the microbiota composition of *T. putrescentiae* populations with and without the presence of the predator (one way PERMANOVA; F = 5.337, P = 0.024) according to Bray-Curtis distance matrix. The random forest algorithm (forest error rate = 0.33) revealed that the following OTUs were important for differences and based on METASTATS were abundant in the microbiome of *T. putrescentiae* from *N. cucumeris* rearing units compared to *T. putrescentiae* pure colonies.: *Staphylococcus saprophyticus* (OTU7), *Brenneria (*OTU9), *Bartonella* (OTU3), *Kocuria koreensis* (OTU25 and OTU39) (Table 1). The Venn diagram (Fig. 4) indicated only 2 OTUs to be unique for *T. putrescentiae* from mass-rearing system (*Leuconostoc gasicomitatum* OTU48) and without predator (*Lactococcus lactis* OTU53). The bacterium similar to *Staphylococcus kloosii* (OTU2) made up a large percentage of the microbiota from both *N. cucumeris* populations and the *T. putrescentiae* population from the mass-rearing where *N. cucumeris* was present.

## Discussion

### Bacterial ecology of *N. cucumeris* and its prey mite *T. putrescentiae*

To our knowledge this is the first study comparing the entire bacterial microbiome of predatory phytoseiid mites and prey astigmatid mites used together under mass rearing conditions. We found that the predatory mite *N. cucumeris* and the prey mite *T. putrescentiae* shared 15% of core bacterial taxa. These taxa differ in relative abundance between predatory and prey mites, and among populations. Similarly, previous studies comparing the bacterial microbiota of *M. occidentalis* and its spider mite prey *Tetranychus urticae* (Koch) (Acari: Tetranychidae), provided evidence for a shared microbiota between the predator and the prey^22^. The dissimilarity in the bacterial community between lab reared and mass-reared populations of *T. putrescentiae* was higher in prey mites than in predatory mites. The prey mites showed higher diversity in their overall microbiota than predatory mites. The differences in diversity of microbiota among saprophagous compared to predatory groups are known in insects^23^ and a similar situation is expected in these mites.

The OTU similar to *S. kloosii* accounted for a large proportion of the total bacterial sequences isolated from both *N. cucumeris* (72% mass-reared, 61% lab-reared) and *T. putrescentiae* populations (40%) the latter from the mass rearing where the predator was present. In the *T. putrescentiae* pure population, where no *N. cucumeris* was present, this bacterium was still present but yet not so prevalent. A similar pattern was seen for other *Staphylococcus* OTUs found in this study. This suggests that bacteria might be transmitted from *N. cucumeris* to its factitious prey, possibly via direct contact or through the feces. Alternatively, the rearing environment of *T. putrescentiae* is not as optimal for *Staphylococcus* growth as the habitat in the presence of the predator. Our suggestion is that the predatory mites can alter the rearing environment in such a way that promotes the proliferation of certain bacterial taxa. Taking into account the pattern observed here as well as the previously demonstrated effects of diet and environment on Astigmata mites microbiota^17,24,25^, it is possible that the microbiota of *T. putrescentiae* is more influenced by diet and environment than by the microbiota of *N. cucumeris*.

The bacterial community of *T. putrescentiae* exhibited higher diversity than that of the predatory mites. Acquisition of bacteria through the prey mites’ diet might explain these results. It is important to highlight that the analyses of differences in the bacterial composition (PCoA) and population level analyses showed that the differences between predatory and prey mites were lower than among the two prey mite populations examined. This is supported by differences between predator and prey in relative numbers of *Blattabacterium*-like and *Solitalea*-like symbiotic bacteria, with a higher relative abundance in prey mites. This suggests that the microbiota of different *T. putrescentiae* populations are more variable than the microbiota of different *N. cucumeris* populations.

While feeding, it is suggested that *T. putrescentiae* ingests debris of plant cells, fungi or yeasts which are already colonized by environmental bacteria^26–28^. The environmental bacteria enter the gut with the food and are passed through the gut or the bacteria adhere to the mite integument in the manner that fungal spores do^29^. Both modes of transmission resulted in the presence of environmental bacteria in the microbiota of astigmatids^17,25^. In the present study the mites were surface sterilized with sterilized phosphate saline buffer and tween 20, thus the identified bacteria species should be regarded as colonizing the mites internally. However, we cannot explicitly exclude either the possibility of some levels of surface contamination or random ingestion of environmental bacteria.

Based on previous studies with stored product mites we can identify the bacterial taxa which could be considered as environmental^17^. For example, *Brevibacterium*, *Staphylococcus*, *Kocuria* and *Stenotrophomonas* are present in mite laboratory habitats e.g. rearing diets and the feces of mites. However, some bacterial taxa isolated in this study (Table S1) are known to belong to either skin or gut communities in other animals. For example, the genus *Corynebacterium* includes species that are widely found in the microbiota of vertebrates^30,31^, *Anaerococcus* and *Finegoldia* spp. are commonly found among microbial skin communities in humans and can occasionally become pathogenic^32^. Bacterial taxa characteristic of mammal gut communities were also found in this study, particularly bacteria of the genera *Escherichia*, *Lactococcus*, *Leuconostoc*, and *Propionibacterium*. The latter genus contains species often found to have beneficial probiotic and nutritional effects within the gut microbiota of many vertebrate hosts such as humans and ruminants^33^. It is possible that *Propionibacterium* has a similar nutritional effect on its mite host, i.e prey mites. Conversely, some *Propionibacterium* species belong to skin microbiota and may cause disease in some cases^34^.

### Endosymbionts in predatory mite and prey populations

The observed microbiota of the predatory mite *N. cucumeris* and the prey mite *T. putrescentiae* consisted of intracellular symbionts as well as gut/environmental bacteria as it is reported for other species of predatory or saprophagous mites^35,36^. The presence of core bacterial taxa in prey and predatory mites poses the question whether these bacteria are autochthonous or allochthonous, i.e. horizontally transmitted from predator to prey. For example, the intracellular symbiont *Wolbachia* has been shown to be transmitted across trophic levels. *Aedes aegypti* (Diptera: Culicidae) larvae infected with the wMelPop strain did not transfer *Wolbachia* to predators, including copepods (Crustacea: Copepoda) and mosquito species^37^. In contrast, *T. putrescentiae* feeding on *Wolbachia* infested corpses of *Drosophila melanogaster* Meigen (Diptera: Drosophilidae) resulted in establishment of *Wolbachia* population in both mites and *Drosophila* and suggested horizontal transfer^38^. Generally, the presence of prey DNA is common in predators. For example, aphid nuclear and mitochondrial DNA had a detectability period longer than 23 hours in the harlequin ladybird *Harmonia axyridis*^39^. Likewise, the bacteria of prey mites and their DNA can be ingested by the predator mites and may be detected in the predator’s digestive tract^13,40,41^. Therefore, the sole detection of bacterial DNA in predators does not necessarily mean that these bacteria form part of the predator’s microbiota, e.g. *Wolbachia* in *Phytoseiulus persimilis*^42^ and *M. occidentalis*^40^ occurs due to infected prey. Presence of bacteria in the predator’s eggs (and concomitant vertical transfer) would unambiguously confirm the bacterial presence in the microbiota^13,25,41^. Further studies screening the presence of symbiotic bacteria in the eggs and other life stages are needed in order to solve the question of autochthonous or allochthonous origin of symbiotic or parasitic bacteria in phytoseiid mites.

The co-occurrence of intracellular symbiotic bacteria such as *Cardinium* and *Wolbachia* in predatory and prey mites is quite common^6,14,22,43,44^. Double infections within the same individuals were common in tetranychid mites^45^. It is likely that similar occurrences are found in *N. cucumeris* and *T. putrescentiae*. It is interesting to mention the presence of the symbiotic *Blattabacterium*-like bacteria found in all populations of mites examined in this study. *Blattabacterium*-like symbionts were recently identified in some *T. putrescentiae* populations^25^. Bayesian analyses of the 16S rRNA gene sequences showed that *Blattabacterium*-like symbionts clustered as a monophyletic lineage. This cluster is outside *Blattabacterium*^46^, *Cand.* Brownia rhizoecola^47^, *Cand.* Uzinora diaspidicola^48^ and *Cand.* Sulcia muelleri^49^. *Blattabacterium* species are obligate endosymbionts found in all cockroaches. Genome sequencing of these bacteria showed that they have a nutritional role in their host including vitamin synthesis and nitrogen recycling^50,51^. Given their similarity to *Blattabacterium*, the bacteria found in *N. cucumeris* and *T. putrescentiae* populations in this study may fulfil a similar role in their host mites. These *Blattabacterium*-like bacteria may be a unique mite specific lineage of *Flavobacterium* and their presence warrants further study to elicit their role in mites. Given that they were more prevalent in *T. putrescentiae* populations and have been previously isolated from this mite^25^, it is likely that this bacterium is found in predatory mites due to ingestion during feeding rather than an obligate interaction.

*Solitalea*-like bacterium was found in the reproductive tract and parenchymal tissues of *A. siro* L. and in five populations of *T. putrescentiae*^24,25^. The clones formed a new distinct cluster separate from *Solitalea* and other genera of the Sphingobacteriaceae family. Based on their localization in the gut, fat body, reproductive tissues and eggs of *A. siro* a symbiotic mode of action has been suggested^24^. *Bartonella*-like bacteria were previously identified in *Dermatophagoides* spp., *A. siro* and *T. putrescentiae*^16,17,52^. However, *Bartonella*-like bacteria were never amplified from eggs, eliminating the possibility of a vertically transmitted symbiont^25^. Herein, *Bartonella*-like bacteria were found in both predatory and prey mites, showing similar proportions to the predator–prey system of *Cheyletus eruditus* (Schrank) (Acarina: Cheyletidae) – *A. siro*^53^. Finally, *Spiroplasma* a commonly known bacteria from some Phytoseiidae^13^ was not isolated from either mite species in this study. The original screening never isolated *Spiroplasma*^13^ from *N. cucumeris.*

In this study we found significant differences in themicrobiome of *T. putrescentiae* from mass rearing units (with predator) and laboratory culture (without predator). The OTUs predominantly responsible for these differences were similar to *S. saprophyticus*, *Brenneria* and *K. koreensis*. These taxa had higher relative abundance in the bacterial microbiota of *T. putrescentiae* from mass rearing units (predator present) compared to laboratory cultures with no predators present. These taxa are suggested as environmental^17^ and we can only speculate that the mass rearing conditions with the presence of predators are more favorable for their development, possibly due to the buildup of feces or husks of predated prey mites. Surprisingly we found *Bartonella*-like bacteria of higher relative abundance in prey mites from the mass rearing units compared to the laboratory cultures. The higher proportion of *Bartonella*-like bacteria in mass rearing culture with the presence of predators may indicate better conditions for *T. putrescentiae*.

### Potential acaropathogens in mite populations

Not known acaropathogenic bacteria were detected in the natural microbiota of the mites examined in this study. Of special interest is the occurrence of *B. cereus* in all samples of predatory and prey mites. Some *Bacillus* taxa such as *B. spahericus* and *B. thuringiensis* (which are not distinguishable by 16S rRNA) are known to be acaropathogenic^54,55^. While *B. cereus* is suggested to be opportunistic on insects, *B. sphaericus*, *B. papillae* and *B. thuringiensis* are known pathogens^56^. *B. cereus* was previously isolated from the feces of a laboratory population of *T. putrescentiae* reared on dog kernels^27^; the identification was confirmed by cloning and sequencing of the motB gene^57^. The addition of *B. cereus* to this mite diet led to a substantial reduction of population growth. Exo-enzymes of this bacterium have been proposed as aiding mite digestion^27^ altogether with inhibition of the population growth of mites suggesting a sort of opportunistic mode of pathogenesis^56^. The presence of *B. cereus* in all mite samples, coupled with the fact that no differences were detected in the microbiota between healthy and sick populations of predatory mites, suggests that symptoms of illness of the predatory mites in the laboratory colony were not caused by bacteria or that the method we used could not detect the acaropathogenic bacteria. Often, some pathogens make up part of the natural microflora of its host and opportunistically cause disease due to subtle changes in environmental factors, host immune system and even microbiota^58^. Presence of disease symptoms in the lab-reared *N. cucumeris* could not be explained by the presence of harmful bacteria. Instead, perhaps it was the result of the lack of beneficial protective microbes as seen in other animals^58^. Alternatively, the disease symptoms in the laboratory population of the predatory mites might have been caused by viruses, fungi or protozoan pathogens^8^ or accumulation of toxic metabolites such as guanine^10^.

### Conclusion

The microbiota of the prey mite *T. putrescentiae* and the predatory mite *N. cucumeris* consisted of core bacterial taxa present in all prey and predatory mite populations.This core microbiota comprised *Wolbachia*, *Cardinium*, *Bartonella*-like, *Blattabacterium*-like, *Solitalea*-like, *Brevibacterium*, *Staphylococcus* spp. and *B. cereus*. Among them *Brevibacterium*, *Staphylococcus* and *Bacillus* were the most abundant in predatory mites, while *Blattabacterium*-like and *Solitalea-*like bacteria were the most abundant in prey mites. Significant differences were detected between the bacterial communities of prey mites without predators and prey mites reared with *N. cucumeris*. *S. saprophyticus*, *Brenneria* and *K. koreensis* were more abundant in the presence of predators. The occurrence of no acaropathogenic bacteria was examined in predatory mite populations with and without disease symptoms. Interestingly, bacterial microbiome of healthy and with disease symptoms predatory mites showed similar diversity, without significant differences between the two groups. This suggests disease symptoms in this case are caused by non-bacterial entities such as other types of microbes, unsuitable environmental conditions or genetic factors. Further study is required to confirm the cause of disease. Ultimately, characterization of predatory mite and prey microbiota may help inform mass rearing practices, but this understanding also required knowledge on the effects of certain microbial taxa on the health of both species of mites. This study can serve to influence the subsequent study of mechanistic studies of the effects of some bacterial taxa on mite hosts of economic importance.

## Materials and Methods

### Origin of mites

The predatory *N. cucumeris* and prey *T. putrescentiae* mites originated from the Biobest rearing facilities in Belgium (Westerlo). *T. putrescentiae* was reared on a mix of yeast flakes, wheat germ and dried yeasts at a ratio 10:10:1 (w/w)^59^ in rearing units of 0.2 L kept at 27 °C and 85% relative humidity (r.h.). *N. cucumeris* was reared on the aforementioned mix plus *T. putrescentiae* (treatment 4 see below) at a ratio of approximately (1:15; *N. cucumeris*: *T. putrescentiae*) in rearing buckets of 5L (mass rearing) or 0.2 L (laboratory population) kept at 25 °C and 70% r.h. The trial included the following treatments: (i) *N. cucumeris* from a mass-production population (ii) *N. cucumeris* from a laboratory population with disease symptoms (slower movement and lighter coloration of the mites); (iii) pure *T. putrescentiae* (with no predators); (iv) *T. putrescentiae* from rearing units with predatory mites. Three samples were examined for treatments i–iii (see below for sample definition), and six samples for treatment iv (Table S2). Predatory and prey mites from treatment 1 were sampled one week after the addition of the prey mites.

### DNA extraction from mites

One sample consisted of about 50 (predator or prey) mites collected in ethanol (90%). Ethanol was removed and samples were washed 3 times in Phosphate Buffered Saline and Tween-20 (PBST). The mites were homogenized in 500 μL of PBST using a Radnoti tissue grinder (Cat No. 440613, Monrovia, CA, USA). Total DNA was extracted using the Wizard® Genomic DNA Purification kit (Promega, Madison, WI, USA) following the manufacturer’s instructions. The extracted DNA was stored at −20 °C until further analyses.

### Amplification, cloning and sequencing

The quality and presence of bacterial DNA in every sample was tested by PCR amplification using eubacterial primers and routinely using protocols^60^. When amplicons were not obtained, the samples were replaced by new samples positive for amplicons. The DNA samples were sent to MR DNA (http://mrzaqqqsadnalab.com, Shallowater, TX, USA). The sequencing of V1-V3 part of 16S rRNA gene was based by the universal primers 27Fmod and 519Rmod in the Illumina MiSeq platform and the bTEFAP® process^61^. The length of the read was 300 bp, the reads were forward and reverse. The sequencing reads were preprocessed in MR DNA to contingencies. The sequences in bioproject PRJNA321085 were deposited in GenBank SRP074673, barcodes and biosample codes are given in Table S2.

### Data analyses and sequence processing

The contingencies were demultiplexed using MR DNA binning software (http://mrdnalab.com), sequences were renamed and trimmed in MOTHUR v. 1.36.1 software^62^ according to the MiSeq standard operation procedure MiSeq SOP^63^. The actual commands used in MOTHUR are available at http://www.mothur.org/wiki/MiSeq_SOP (accession date 2/19/2016). The demultiplexed, renamed and trimmed sequences were processed in UPARSE and USEARCH^64^, singletons were removed and the sequences were classified using a naive Bayesian classifier with a training set (version 15) made available through the Ribosomal Database Project (RDP) (http://rdp.cme.msu.edu)^65^. Operational taxonomic units (OTUs) were defined by the clustering of sequences at ≥97% identity. The representative sequences obtained from UPARSE were processed in MOTHUR again, aligned against SILVA reference database^66^. The aligned sequences were screened for chimeras with UCHIME^67^. Sequences from chloroplasts, mitochondria, Archaea and Eukaryotes were removed. The representative sequences were analyzed via BLASTin GenBank^68,69^. *Solitalea*-like, *Blattabacterium*-like and *Cardinium* bacteria were identified by aligning OTUs to previously identified almost full length 16S rRNA sequences of these taxa^17,24,25^ in Codone Code Aligner (CodonCode Corporation, Centerville, MA, USA). Taxonomic diversity and relative proportions of bacterial taxa were visualized using Krona tools^70^.

### Statistical analyses

The shared file was generated from UPARSE data and was processed in MOTHUR, and PAST 3.06^71^. The results were visualized by XLSTAT (http://www.xlstat.com/en/, Addinsoft, New York, NY, USA). The subsample data set was constructed in MOTHUR on 17,613 sequences. Alpha-diversity, (OTUs richness) in predatory or prey mites was assessed using the Inverse-Simpson index, the number of OTUs was calculated in MOTHUR from a subsample data set. The Inverse-Simpson index and species level OTUs (sOTU) of the predator and prey samples were compared using a nonparametric Mann-Whitney test. Beta-diversity, (similarity of samples) was assessed using the Bray-Curtis and Jacquard indices and visualized by principal coordinate analyses (PCoA) of the subsample data set. The contribution of OTUs was calculated using Pearson correlation coefficient. We analyzed the effects of two factors on bacterial diversity: (i) mite species (i.e. prey and predator) and (ii) origin of mite population (i.e predator with and without sick symptoms and prey from mass rearing facility and without any predator) (Table S2). The effects of both factors were evaluated with two-way PERMANOVA^72^ with 100,000 permutations. Calculations were based on Bray-Curtis and Jaccard matrices of the subsample data. The different populations of predatory mite (sick and without disease symptoms) and *T. putrescentiae* (from mass production and without predatory mites) were also compared separately. Venn diagrams were used to highlight shared bacterial taxa in mites from different treatments, to the analyses we included only those OTUs, which were presented in all three replicates per treatment. The population-level analysis was calculated using METASTATS^73^ based on 100,000 permutations to compare the effects predator/prey to distribution of bacterial taxa in the subsample data set. The random forest analysis in MOTHUR was applied to compare the differences in bacterial microbiome of sick and healthy predator or *T. putrescentiae* from mass rearing and laboratory culture.

## Electronic supplementary material

Supplementary Tables S1 and S2
