# Author Correction: Comparison of bacterial microbiota of the predatory mite *Neoseiulus cucumeris* (Acari: Phytoseiidae) and its factitious prey *Tyrophagus putrescentiae* (Acari: Acaridae)

**DOI:** 10.1038/s41598-018-19514-8

**Published:** 2018-01-17

**Authors:** Apostolos Pekas, Eric Palevsky, Jason C. Sumner, M. Alejandra Perotti, Marta Nesvorna, Jan Hubert

**Affiliations:** 1 Research & Development Department, Biobest Belgium N. V., Ilse Velden 18, Westerlo B-2260 Belgium; 2 Department of Entomology, Newe-Ya’ar Research Center, Agricultural Research Organization, Ministry of Agriculture, P.O. Box 1021 Ramat Yishay, IL-30095 Israel; 3 SASA (Science and Advice for Scottish Agriculture), 1 Roddinglaw Road, Edinburgh, EH12 9FJ UK; 4 Evolutionary Biology Section, School of Biological Sciences, University of Reading, Reading, RG6 6AS UK; 5 Crop Research Institute, Drnovska 507/73, Prague 6-Ruzyne, CZ-161 06 Czechia Czech Republic

Correction to: *Scientific Reports* 10.1038/s41598-017-00046-6, published online 31 January 2017

The original version of this Article contained an error in Affiliation 3 which was incorrectly listed as ‘SASA (Science and Advice for Scottish Agriculture), 1 Roddinglaw Road, Edinburgh, EH9 2BR, UK’.

The correct affiliation is listed below:

‘SASA (Science and Advice for Scottish Agriculture), 1 Roddinglaw Road, Edinburgh, EH12 9FJ, UK’.

This error has now been corrected in the HTML and PDF versions of the Article.

