# Supplementary material for: Comparison of bacterial microbiota of the predatory mite Neoseiulus cucumeris (Acari: Phytoseiidae) and its factitious prey Tyrophagus putrescentiae (Acari: Acaridae)
Source: Sci Rep. 2017 Jan 31;7:2. doi: 10.1038/s41598-017-00046-6 (PMC5428342; doi:10.1038/s41598-017-00046-6)
Supplement: Supplementary file 1 — Supplementary Tables S1 and S2 [file 41598_2017_46_MOESM1_ESM.pdf]

Supplementary material

Pekas A, Palevsky E, Sumner JC, Perotti AM, Nesvorna M, Hubert J.

Comparison of bacterial microbiota of the predatory mite *Neoseiulus cucumeris* (Acari: Phytoseiidae) and its factitious prey *Tyrophagus putrescentiae* (Acari: Acaridae)

Scientific Reports

Table S1. List of OTUs97 in the samples of prey (Tyrophagus putrescentiae) and predatory (Neoseiulus cucumeris) mites. Classification of OTUs up to genus level was performed using the classifier tool from RDP (https://rdp.cme.msu.edu/classifier/classifier.jsp). Confidence thresholds are shown in parentheses. Closest species matches are according to BLAST analysis of the GenBank database (http://www.ncbi.nlm.nih.gov/genbank/), percentage identity is provided in parentheses. Proportional representation of sequences belonging to each bacterial species is also shown. Key: Red, not present; White, <0.001, Pale Blue, 0.001-0.3; Light Blue, >0.3-0.5; Dark Blue >0.5. The colors in the table are the markers for numbers of sequences.

	RDP -identification					GenBank		Numbers			Predatory mite samples						Prey mite samples								
**OTU**	**phylum**	**class**	**order**	**family**	**genus**	**Taxa**	**Aces. No.**	**Total**	**Predator**	**Prey**	**BB1**	**BB2**	**BB3**	**BB4**	**BB5**	**BB6**	**Tyro1**	**Tyro2**	**Tyro3**	**Tyro4**	**Tyro5**	**Tyro6**	**Tyro7**	**Tyro8**	**Tyro9**
OTU1	Proteobacteria(0.9990)	Alphaproteobacteria(0.7029)	Rickettsiales(0.3260)	Anaplasmataceae(0.1514)	Anaplasma(0.0173)	Wolbachia sp. wRi(97)	NR_074437	128107	12275	115832	0.017	0.027	0.038	0.071	0.178	0.045	0.644	0.058	0.412	0.036	0.016	0.069	0.234	0.004	0.345
OTU101	Actinobacteria(0.9998)	Actinobacteria(0.9977)	Actinomycetales(0.9757)	Brevibacteriaceae(0.8530)	Brevibacterium(0.6783)	Brevibacterium siliguriense(96)	NR_115086	24	24	0	-	0.001	-	0.000	0.000	-	-	-	-	-	-	-	-	-	-
OTU102	Proteobacteria(0.9994)	Gammaproteobacteria(0.9773)	Oceanospirillales(0.8226)	Alcanivoracaceae(0.6207)	Alcanivorax(0.4313)	Alcanivorax dieselolei(94)	NR_074734	20	1	19	-	-	-	-	-	0.000	-	-	-	-	0.000	0.000	-	-	-
OTU103	Proteobacteria(1.0000)	Gammaproteobacteria(0.9990)	Enterobacteriales(0.9832)	Enterobacteriaceae(0.9854)	Escherichia/Shigella(0.7790)	Escherichia coli(99)	NR_102804	23	2	21	-	-	0.000	-	-	-	-	-	0.000	0.000	-	0.000	0.000	-	0.000
OTU104	Actinobacteria(1.0000)	Actinobacteria(0.9988)	Actinomycetales(0.9870)	Corynebacteriaceae(0.9691)	Corynebacterium(0.9756)	Corynebacterium singulare(99)	NR_026394	18	18	0	0.000	-	-	-	0.000	-	-	-	-	-	-	-	-	-	-
OTU11	Proteobacteria(0.9878)	Alphaproteobacteria(0.6351)	Rickettsiales(0.2560)	Anaplasmataceae(0.1157)	Anaplasma(0.0148)	Wolbachia endosymbiont(97)	NR_074127	667	334	333	-	-	-	0.002	0.007	0.002	0.000	-	0.000	-	-	0.000	0.003	-	0.000
OTU12	Bacteroidetes(0.8102)	Cytophagia	Cytophagales	Cytophagales	Cytophagales	Cardinium	nd	1398	530	868	0.002	0.003	0.002	0.002	0.001	0.002	0.001	0.004	0.001	0.002	0.002	0.002	0.001	0.001	0.001
OTU13	Proteobacteria(0.9699)	Alphaproteobacteria(0.4993)	Rickettsiales(0.2209)	Anaplasmataceae(0.1006)	Anaplasma(0.0123)	Wolbachia sp. wRi(99)	NR_074437	678	667	11	0.011	-	-	-	-	-	0.000	0.000	-	-	0.000	0.000	0.000	-	0.000
OTU16	Actinobacteria(1.0000)	Actinobacteria(0.9985)	Actinomycetales(0.9851)	Brevibacteriaceae(0.9309)	Brevibacterium(0.9132)	Brevibacterium siliguriense(97)	NR_115086	1273	679	594	0.002	0.004	0.001	0.001	0.011	0.002	0.000	0.001	0.000	0.001	0.003	0.001	0.001	0.004	0.000
OTU18	Actinobacteria(1.0000)	Actinobacteria(0.9988)	Actinomycetales(0.9890)	Brevibacteriaceae(0.9462)	Brevibacterium(0.9693)	Brevibacterium oceani(99)	NR_042458	544	464	80	0.001	0.002	-	0.000	0.003	0.006	-	0.000	-	0.000	0.001	-	0.000	0.000	-
OTU19	Firmicutes(1.0000)	Bacilli(0.9992)	Bacillales(0.9903)	Staphylococcaceae(0.9861)	Staphylococcus(0.9881)	Staphylococcus cohnii(99)	NR_037046	4654	1993	2661	0.008	0.010	0.008	0.009	0.006	0.007	0.001	0.005	0.001	0.007	0.008	0.007	0.004	0.006	0.002
OTU2	Firmicutes(1.0000)	Bacilli(0.9973)	Bacillales(0.9929)	Staphylococcaceae(0.9903)	Staphylococcus(0.9957)	Staphylococcus kloosii(99)	NR_024667	389716	180425	209291	0.764	0.750	0.774	0.749	0.492	0.716	0.004	0.001	0.001	0.712	0.734	0.612	0.242	0.727	0.033
OTU21	Firmicutes(1.0000)	Bacilli(0.9994)	Bacillales(0.9890)	Bacillaceae_1(0.9833)	Bacillus(0.9798)	Bacillus cereus(99)	NR_074540	1995	836	1159	0.004	0.004	0.004	0.003	0.003	0.003	0.001	0.003	0.001	0.003	0.003	0.002	0.002	0.003	0.001
OTU22	Actinobacteria(0.9993)	Actinobacteria(0.9951)	Actinomycetales(0.9700)	Corynebacteriaceae(0.8142)	Corynebacterium(0.6448)	Corynebacterium variabile(97)	NR_102874	279	126	153	-	-	-	0.001	0.002	0.001	-	-	0.000	0.000	0.001	0.001	0.000	0.000	0.000
OTU23	Firmicutes(1.0000)	Bacilli(0.9998)	Bacillales(0.9995)	Staphylococcaceae(0.9875)	Staphylococcus(0.9923)	Staphylococcus lentus(99)	NR_043418	466	251	215	0.002	0.001	0.001	0.001	0.002	0.001	0.000	-	0.000	0.001	0.000	0.000	0.001	0.001	0.000
OTU25	Actinobacteria(0.9998)	Actinobacteria(0.9979)	Actinomycetales(0.9719)	Micrococcaceae(0.8928)	Kocuria(0.5777)	Kocuria koreensis(96)	NR_116745	286	192	94	0.000	0.001	0.000	0.000	0.002	0.002	0.000	-	-	0.000	0.000	0.000	0.000	0.000	0.000
OTU26	Actinobacteria(1.0000)	Actinobacteria(0.9987)	Actinomycetales(0.9886)	Brevibacteriaceae(0.9462)	Brevibacterium(0.9693)	Brevibacterium iodinum(98)	NR_026241	325	175	150	0.001	0.001	-	0.000	0.003	0.000	-	-	-	0.000	0.000	0.000	0.000	0.001	0.000
OTU3	Proteobacteria(0.9994)	Alphaproteobacteria(0.9628)	Rhizobiales(0.6947)	Bartonellaceae(0.4269)	Bartonella(0.1766)	Bartonella queenslandensis (95)	NR_116176	75065	10011	65054	0.032	0.043	0.041	0.036	0.045	0.051	0.131	0.588	0.216	0.045	0.017	0.048	0.125	0.002	0.170
OTU31	Actinobacteria(0.9997)	Actinobacteria(0.9976)	Actinomycetales(0.9912)	Propionibacteriaceae(0.9896)	Propionibacterium(0.9956)	Propionibacterium acnes(99)	NR_074675	147	36	111	0.000	0.000	0.000	0.000	0.000	0.000	0.000	-	0.000	0.000	0.001	0.000	0.000	0.000	-
OTU32	Proteobacteria(0.9996)	Alphaproteobacteria(0.9773)	Rhizobiales(0.8634)	Brucellaceae(0.5433)	Ochrobactrum(0.3294)	Bartonella coopersplainsensis(95)	NR_116177	81	32	49	0.000	0.000	0.000	0.000	0.000	0.000	0.000	0.000	0.000	0.000	0.000	0.000	0.000	0.000	0.000
OTU34	Actinobacteria(0.9993)	Actinobacteria(0.9962)	Actinomycetales(0.9832)	Microbacteriaceae(0.9462)	Microbacterium(0.8126)	Microbacterium indicum(97)	NR_042459	107	31	76	-	0.000	-	-	0.001	-	-	-	-	-	0.002	0.000	0.000	-	-
OTU35	Proteobacteria(0.9997)	Gammaproteobacteria(0.9968)	Pseudomonadales(0.9895)	Moraxellaceae(0.9889)	Enhydrobacter(0.9777)	Moraxella osloensis(99)	NR_113392	138	109	29	0.001	0.000	0.001	0.000	-	0.000	0.000	0.000	0.000	-	0.000	0.000	0.000	-	-
OTU36	Proteobacteria(1.0000)	Alphaproteobacteria(0.9961)	Rhodobacterales(0.9832)	Rhodobacteraceae(0.9854)	Paracoccus(0.8126)	Paracoccus chinensis(99)	NR_114276	72	71	1	0.001	-	-	-	-	-	-	-	-	-	-	-	0.000	-	-
OTU38	Proteobacteria(1.0000)	Alphaproteobacteria(0.9956)	Rhizobiales(0.9700)	Bartonellaceae(0.8928)	Bartonella(0.7454)	Bartonella rattaustraliani(98)	NR_116175	45	11	34	0.000	0.000	0.000	0.000	-	0.000	0.000	0.000	0.000	0.000	0.000	0.000	0.000	0.000	0.000
OTU39	Actinobacteria(0.9997)	Actinobacteria(0.9974)	Actinomycetales(0.9916)	Micrococcaceae(0.9861)	Kocuria(0.9818)	Kocuria koreensis(99)	NR_116745	211	128	83	0.001	0.000	0.000	0.000	0.001	0.001	0.000	-	-	0.000	0.000	0.000	0.000	0.000	0.000
OTU40	Actinobacteria(0.9999)	Actinobacteria(0.9980)	Actinomycetales(0.9775)	Corynebacteriaceae(0.8928)	Corynebacterium(0.7454)	Corynebacterium nuruki(99)	NR_117816	64	27	37	0.000	0.001	-	-	-	0.000	-	-	-	-	0.001	0.000	0.000	-	0.000
OTU42	Bacteroidetes(0.9995)	Bacteroidia(0.9943)	Bacteroidales(0.9719)	Prevotellaceae(0.9080)	Prevotella(0.7790)	Prevotella paludivivens(97)	NR_113122	73	72	1	-	-	0.002	-	-	-	-	0.000	-	-	-	-	-	-	-
OTU43	Proteobacteria(0.9992)	Gammaproteobacteria(0.9700)	Enterobacteriales(0.8226)	Enterobacteriaceae(0.6207)	Xenorhabdus(0.0788)	Xenorhabdus innexi(92)	NR_042325	221	41	180	0.000	0.000	0.000	0.000	0.000	0.000	0.000	0.000	0.000	0.000	0.000	0.000	0.001	0.000	0.000
OTU45	Proteobacteria(0.9991)	Alphaproteobacteria(0.9065)	Rickettsiales(0.4815)	Anaplasmataceae(0.3088)	Anaplasma(nd)	Wolbachia endosymbiont(94)	NR_074571	97	28	69	0.000	0.000	0.000	0.000	0.000	0.000	0.000	0.000	0.000	0.000	0.000	0.000	0.000	0.000	0.000
OTU47	Actinobacteria(0.9994)	Actinobacteria(0.9700)	Actinomycetales(0.8849)	Micrococcaceae(0.4269)	Arthrobacter(0.0897)	Arthrobacter russicus(91)	NR_024783	66	30	36	0.000	0.000	-	0.000	0.000	0.000	0.000	0.000	0.000	0.000	0.000	0.000	0.000	0.000	0.000
OTU48	Firmicutes(1.0000)	Bacilli(0.9998)	Lactobacillales(0.9995)	Leuconostocaceae(0.9934)	Leuconostoc(0.9902)	Leuconostoc gasicomitatum(99)	NR_074997	69	19	50	-	-	-	0.000	-	0.000	-	-	-	0.000	0.001	0.000	-	0.000	-
OTU49	Proteobacteria(1.0000)	Gammaproteobacteria(0.9970)	Pseudomonadales(0.9890)	Moraxellaceae(0.9882)	Acinetobacter(0.9923)	Acinetobacter baumannii(99)	NR_117677	70	70	0	0.001	-	0.000	0.000	0.001	-	-	-	-	-	-	-	-	-	-
OTU5	Bacteroidetes(0.5587)	Flavobacteriia(0.0162)	Flavobacteriales(0.0054)	Flavobacteriaceae(0.0023)	Tenacibaculum(0.0025)	Blattabacterium-like	nd	76261	3298	72963	0.010	0.007	0.007	0.007	0.028	0.026	0.118	0.008	0.189	0.012	0.010	0.091	0.145	0.135	0.309
OTU50	Proteobacteria(1.0000)	Gammaproteobacteria(0.9998)	Pseudomonadales(0.9907)	Pseudomonadaceae(0.9910)	Pseudomonas(0.9818)	Pseudomonas poae(99)	NR_102514	77	5	72	0.000	0.000	-	-	-	-	-	-	-	-	-	-	0.001	-	-
OTU51	Proteobacteria(1.0000)	Betaproteobacteria(0.9960)	Burkholderiales(0.9832)	Alcaligenaceae(0.9854)	Alcaligenes(0.9132)	Alcaligenes faecalis(99)	NR_113606	115	20	95	0.000	-	-	-	0.001	-	0.000	-	-	0.001	-	0.000	-	-	0.000
OTU52	Proteobacteria(1.0000)	Gammaproteobacteria(0.9969)	Xanthomonadales(0.9170)	Xanthomonadaceae(0.8928)	Stenotrophomonas(0.7454)	Stenotrophomonas maltophilia(99)	NR_113648	71	46	25	0.000	-	0.000	0.000	0.000	0.000	-	-	0.000	0.000	-	0.000	0.000	0.000	-
OTU53	Firmicutes(0.9997)	Bacilli(0.9975)	Lactobacillales(0.9929)	Streptococcaceae(0.9903)	Lactococcus(0.9957)	Lactococcus lactis(99)	NR_074949	84	48	36	0.001	0.000	-	0.000	-	-	0.000	0.000	0.000	-	-	-	0.000	0.000	0.000
OTU54	Proteobacteria(1.0000)	Gammaproteobacteria(0.9969)	Pseudomonadales(0.9886)	Pseudomonadaceae(0.9875)	Pseudomonas(0.9798)	Pseudomonas caeni (99)	NR_116388	25	17	8	-	0.000	0.000	-	-	-	-	-	-	-	0.000	-	-	-	-
OTU55	Proteobacteria(1.0000)	Gammaproteobacteria(0.9961)	Pseudomonadales(0.9813)	Moraxellaceae(0.9840)	Acinetobacter(0.9798)	Acinetobacter lwoffii(97)	NR_026209	103	99	4	0.001	0.001	0.000	-	0.000	-	0.000	-	-	-	-	-	0.000	-	-
OTU56	Firmicutes(0.9993)	Bacilli(0.9953)	Lactobacillales(0.9757)	Streptococcaceae(0.9386)	Lactococcus(0.9132)	Lactococcus chungangensis(97)	NR_044357	31	30	1	0.000	0.000	-	-	-	-	-	-	-	-	-	-	0.000	-	-
OTU57	Proteobacteria(1.0000)	Alphaproteobacteria(0.9966)	Rhizobiales(0.9870)	Brucellaceae(0.9386)	Paenochrobactrum(0.8126)	Paenochrobactrum glaciei(99)	NR_112750	38	0	38	-	-	-	-	-	-	-	-	-	0.000	-	-	0.000	-	0.000
OTU6	Bacteroidetes(0.9341)	Sphingobacteriia(0.2957)	Sphingobacteriales(0.1509)	Sphingobacteriales(0.1509)	Sphingobacteriales(0.1509)	Solitalea-like	nd	54621	7409	47212	0.020	0.026	0.062	0.022	0.048	0.024	0.096	0.331	0.177	0.034	0.074	0.043	0.082	0.003	0.102
OTU60	Bacteroidetes(0.9995)	Flavobacteriia(0.9961)	Flavobacteriales(0.9890)	Flavobacteriaceae(0.9882)	Wautersiella(0.6448)	Wautersiella falsenii(97)	NR_042430	78	64	14	0.001	-	-	0.000	0.001	0.000	-	-	-	-	-	-	0.000	0.000	0.000
OTU61	Proteobacteria(1.0000)	Gammaproteobacteria(0.9994)	Pseudomonadales(0.9890)	Pseudomonadaceae(0.9882)	Pseudomonas(0.9798)	Pseudomonas monteilii(99)	NR_121767	149	131	18	0.001	0.002	0.000	0.000	-	0.000	0.000	0.000	0.000	-	-	-	0.000	-	0.000
OTU62	Bacteroidetes(0.9999)	Flavobacteriia(0.9955)	Flavobacteriales(0.9813)	Flavobacteriaceae(0.9847)	Cloacibacterium(0.7454)	Cloacibacterium rupense(96)	NR_114274	36	0	36	-	-	-	-	-	-	-	-	-	-	-	-	-	0.001	-
OTU63	Proteobacteria(1.0000)	Gammaproteobacteria(0.9961)	Pseudomonadales(0.9775)	Moraxellaceae(0.9833)	Acinetobacter(0.9756)	Acinetobacter johnsonii(99)	NR_117624	56	51	5	0.000	-	0.001	0.000	-	-	-	-	-	0.000	-	-	0.000	-	-
OTU64	Proteobacteria(1.0000)	Alphaproteobacteria(0.9995)	Caulobacterales(0.9890)	Caulobacteraceae(0.9882)	Brevundimonas(0.9468)	Brevundimonas bullata(99)	NR_025831	21	17	4	0.000	0.000	-	-	-	-	-	-	-	-	-	-	-	-	0.000
OTU65	Proteobacteria(1.0000)	Alphaproteobacteria(0.9964)	Rhizobiales(0.9813)	Phyllobacteriaceae(0.9233)	Phyllobacterium(0.8461)	Phyllobacterium myrsinacearum	NR_113874	4	1	3	-	-	-	-	-	0.000	-	-	-	-	0.000	-	-	-	-
OTU68	Bacteroidetes(0.9995)	Flavobacteriia(0.9870)	Flavobacteriales(0.9662)	Flavobacteriaceae(0.9386)	Chryseobacterium(0.2276)	Chryseobacterium bernardetii(94)	NR_126254	11	0	11	-	-	-	-	-	-	-	-	-	-	-	-	-	0.000	-
OTU69	Actinobacteria(0.9999)	Actinobacteria(0.9980)	Actinomycetales(0.9738)	Corynebacteriaceae(0.9004)	Corynebacterium(0.7790)	Corynebacterium vitaeruminis(99)	NR_121721	32	0	32	-	-	-	-	-	-	-	-	-	0.000	-	-	-	0.001	-
OTU7	Firmicutes(1.0000)	Bacilli(0.9998)	Bacillales(0.9995)	Staphylococcaceae(0.9896)	Staphylococcus(0.9954)	Staphylococcus saprophyticus(99)	NR_074999	53443	24367	29076	0.115	0.113	0.055	0.091	0.144	0.094	0.000	0.000	0.000	0.077	0.117	0.086	0.041	0.109	0.007
OTU70	Proteobacteria(1.0000)	Alphaproteobacteria(0.9995)	Rhizobiales(0.9794)	Brucellaceae(0.9615)	Pseudochrobactrum(0.9714)	Pseudochrobactrum asaccharolyticum(99	NR_042474	36	5	31	-	-	-	-	0.000	-	-	-	-	-	-	-	0.000	-	0.000
OTU71	Proteobacteria(1.0000)	Gammaproteobacteria(0.9998)	Enterobacteriales(0.9890)	Enterobacteriaceae(0.9882)	Leclercia(0.7454)	Leclercia adecarboxylata(99)	NR_114154	44	43	1	0.000	0.001	-	-	-	-	-	-	-	-	-	-	0.000	-	-
OTU72	Firmicutes(0.9995)	Clostridia(0.9966)	Clostridiales(0.9907)	Clostridiales_Incertae_Sedis_XI(0.9882)	Finegoldia(0.9881)	Finegoldia magna(99)	NR_113383	18	18	0	-	0.000	0.000	-	-	-	-	-	-	-	-	-	-	-	-
OTU74	Bacteroidetes(1.0000)	Sphingobacteriia(0.9953)	Sphingobacteriales(0.9794)	Sphingobacteriaceae(0.9840)	Sphingobacterium(0.9818)	Sphingobacterium faecium(99)	NR_113744	21	20	1	0.000	-	-	-	-	-	-	-	-	-	-	-	0.000	-	-
OTU75	Proteobacteria(1.0000)	Betaproteobacteria(0.9965)	Burkholderiales(0.9890)	Comamonadaceae(0.9539)	Acidovorax(0.8461)	Acidovorax radicis(99)	NR_117776	13	10	3	-	0.000	-	0.000	-	-	-	-	-	-	0.000	-	-	-	-
OTU77	Proteobacteria(1.0000)	Betaproteobacteria(0.9960)	Burkholderiales(0.9851)	Burkholderiaceae(0.9833)	Ralstonia(0.8797)	Ralstonia insidiosa(99)	NR_025242	47	21	26	-	-	0.000	-	-	0.000	0.000	-	0.000	-	0.000	-	-	-	-
OTU79	Proteobacteria(1.0000)	Betaproteobacteria(0.9968)	Burkholderiales(0.9899)	Comamonadaceae(0.9847)	Delftia(0.9735)	Delftia tsuruhatensis(99)	NR_113870	26	18	8	-	0.000	0.000	-	0.001	-	0.000	-	-	-	-	-	0.000	-	-
OTU80	Proteobacteria(1.0000)	Alphaproteobacteria(0.9966)	Rhodobacterales(0.9870)	Rhodobacteraceae(0.9868)	Paracoccus(0.9468)	Paracoccus marinus(99)	NR_113921	10	10	0	-	-	0.000	-	-	-	-	-	-	-	-	-	-	-	-
OTU81	Bacteroidetes(1.0000)	Flavobacteriia(0.9985)	Flavobacteriales(0.9966)	Flavobacteriaceae(0.9963)	Chryseobacterium(0.8126)	Chryseobacterium balustinum(97)	NR_113721	9	9	0	0.000	-	-	-	-	-	-	-	-	-	-	-	-	-	-
OTU82	Proteobacteria(1.0000)	Gammaproteobacteria(0.9964)	Pseudomonadales(0.9851)	Moraxellaceae(0.9854)	Acinetobacter(0.9818)	Acinetobacter radioresistens (99)	NR_114074	22	14	8	0.000	0.000	-	-	-	-	-	-	-	-	-	-	0.000	-	0.000
OTU83	Proteobacteria(0.9996)	Gammaproteobacteria(0.9894)	Enterobacteriales(0.8849)	Enterobacteriaceae(0.8142)	Pantoea(0.3804)	Pantoea calida (94)	NR_117304	10	8	2	0.000	-	-	-	-	-	0.000	-	-	-	-	-	-	-	-
OTU84	Proteobacteria(1.0000)	Gammaproteobacteria(0.9968)	Xanthomonadales(0.9599)	Xanthomonadaceae(0.9233)	Stenotrophomonas(0.8797)	Stenotrophomonas rhizophila(99)	NR_121739	12	12	0	0.000	0.000	-	0.000	-	-	-	-	-	-	-	-	-	-	-
OTU86	Firmicutes(1.0000)	Bacilli(0.9994)	Lactobacillales(0.9907)	Lactobacillaceae(0.9875)	Lactobacillus(0.9902)	Lactobacillus paracollinoides(99)	NR_112846	7	6	1	-	-	0.000	-	-	-	0.000	-	-	-	-	-	-	-	-
OTU87	Bacteroidetes(1.0000)	Sphingobacteriia(0.9957)	Sphingobacteriales(0.9851)	Sphingobacteriaceae(0.9861)	Sphingobacterium(0.9881)	Sphingobacterium multivorum(99)	NR_040953	59	14	45	0.000	-	-	-	-	-	-	-	0.000	0.000	-	-	0.000	-	-
OTU88	Proteobacteria(1.0000)	Alphaproteobacteria(0.9960)	Rickettsiales(0.9870)	Rickettsiaceae(0.9868)	Rickettsia(0.9818)	Rickettsia bellii(99)	NR_074484	7	0	7	-	-	-	-	-	-	-	-	-	-	-	-	0.000	-	-
OTU89	Proteobacteria(0.9993)	Gammaproteobacteria(0.9959)	Enterobacteriales(0.9757)	Enterobacteriaceae(0.9768)	Buchnera(0.9756)	Buchnera aphidicola(97)	NR_074512	12	11	1	-	0.000	-	0.000	-	-	-	-	0.000	-	-	-	-	-	-
OTU9	Proteobacteria(0.9994)	Gammaproteobacteria(0.9628)	Enterobacteriales(0.5241)	Enterobacteriaceae(0.3482)	Brenneria(0.0571)	Brenneria salicis(91)	NR_114714	25625	1408	24217	0.000	0.000	0.000	0.002	0.017	0.018	0.000	0.000	0.000	0.066	0.005	0.037	0.117	0.001	0.025
OTU91	Firmicutes(0.9996)	Bacilli(0.9973)	Lactobacillales(0.9920)	Streptococcaceae(0.9917)	Streptococcus(0.9960)	Streptococcus thermophilus(99)	NR_074827	36	35	1	0.000	0.000	0.000	-	-	-	-	-	-	-	-	-	0.000	-	-
OTU92	Firmicutes(1.0000)	Bacilli(0.9957)	Bacillales(0.9832)	Paenibacillaceae_1(0.9615)	Paenibacillus(0.9132)	Paenibacillus hordei (97)	NR_109318	13	0	13	-	-	-	-	-	-	-	-	-	-	0.000	-	-	-	-
OTU93	Proteobacteria(1.0000)	Alphaproteobacteria(0.9970)	Rhizobiales(0.9886)	Bradyrhizobiaceae(0.9080)	Afipia(0.7790)	Afipia birgiae (99)	NR_025117	7	4	3	-	0.000	-	-	-	-	-	-	-	-	-	-	-	-	0.000
OTU94	Firmicutes(1.0000)	Bacilli(0.9998)	Bacillales(0.9995)	Staphylococcaceae(0.9903)	Staphylococcus(0.9944)	Staphylococcus aureus(99)	NR_075000	49	15	34	0.000	0.000	0.000	0.000	0.000	-	-	-	-	-	0.001	-	0.000	-	-
OTU95	Actinobacteria(0.9999)	Actinobacteria(0.9982)	Actinomycetales(0.9794)	Microbacteriaceae(0.8928)	Leucobacter(0.6448)	Leucobacter denitrificans(97)	NR_108568	21	1	20	-	-	-	-	0.000	-	-	-	-	0.000	-	-	0.000	-	0.000
OTU96	Actinobacteria(1.0000)	Actinobacteria(0.9998)	Actinomycetales(0.9920)	Tsukamurellaceae(0.9615)	Tsukamurella(0.9735)	Tsukamurella paurometabola(99)	NR_074458	48	23	25	0.000	0.000	0.000	0.000	-	0.000	-	0.000	0.000	0.000	-	0.000	0.000	0.000	0.000
OTU97	Firmicutes(0.9993)	Clostridia(0.9919)	Clostridiales(0.9700)	Clostridiales_Incertae_Sedis_XI(0.9386)	Anaerococcus(0.9468)	Anaerococcus senegalensis(99)	NR_118220	6	6	0	-	0.000	-	-	-	-	-	-	-	-	-	-	-	-	-
**N**								818,413	247,022	571,391	**61,868**	**27,512**	**35,197**	**48,610**	**17,881**	**55,954**	**41,229**	**17,613**	**62,970**	**88,034**	**34,069**	**88,633**	**111,256**	**51,677**	**75,910**

Legend: nd -not determined

Table S2. The description of the samples of predatory (Neoseiulus cucumeris) and prey (Tyrophagus putrescentiae) mites and the variables tested in the study. The barcodes and samples ID in GenBank are provided. The sequences were deposited in SRA SRP074673 as bioproject PRJNA321085 in GenBank.

Sample code	Date collected	Species	Description	factor1	factor2	Sample ID.	Barcodes
BB1	3/26/2015	*N. cucumeris*	Mass-production population	pred	1	SAMN04959061	CTCGACGA
BB2	3/26/2015	*N. cucumeris*	Mass-production population	pred	1	SAMN04959062	CTCGTACG
BB3	3/26/2015	*N. cucumeris*	Mass-production population	pred	1	SAMN04959063	CTCGTGAT
BB4	3/31/2015	*N. cucumeris*	Lab population with disease symptoms	pred	2	SAMN04959064	CTCTAGCG
BB5	3/31/2015	*N. cucumeris*	Lab population with disease symptoms	pred	2	SAMN04959065	CTCTATTT
BB6	3/31/2015	*N. cucumeris*	Lab population with disease symptoms	pred	2	SAMN04959066	CTGCGTTG
Tyro 1	3/31/2015	*T. putrescentiae*	Pure culture (no *N. cucumeris*)	prey	3	SAMN04959080	CTCTGTGT
Tyro 2	3/31/2015	*T. putrescentiae*	Pure culture (no *N. cucumeris*)	prey	3	SAMN04959072	CTGAATCA
Tyro 3	3/31/2015	*T. putrescentiae*	Pure culture (no *N. cucumeris*)	prey	3	SAMN04959073	CTGACCCG
Tyro 4	3/31/2015	*T. putrescentiae*	Mites from N. cucumeris mass-production	prey	4	SAMN04959074	CTGACGGC
Tyro 5	3/31/2015	*T. putrescentiae*	Mites from N. cucumeris mass-production	prey	4	SAMN04959075	CTGAGCGT
Tyro 6	3/31/2015	*T. putrescentiae*	Mites from N. cucumeris mass-production	prey	4	SAMN04959076	CTGATAAC
Tyro 7	3/31/2015	*T. putrescentiae*	Mites from N. cucumeris mass-production	prey	4	SAMN04959077	CTGATAGC
Tyro 8	3/31/2015	*T. putrescentiae*	Mites from N. cucumeris mass-production	prey	4	SAMN04959078	CTGCCGCC
Tyro 9	3/31/2015	*T. putrescentiae*	Mites from N. cucumeris mass-production	prey	4	SAMN04959079	CTGCGAGG
